# Remarks upon Bonwill's Method of Restoring Crowns upon Roots

**Published:** 1882-01

**Authors:** E. A. Stebbins

**Affiliations:** Shelburne Falls, Mass.


### ARTICLE VI.

### *Remarks upon Bonwill's Method of Restoring Crowns upon Roots.*

BY DR. E. A. STEBBINS, SHELBURNE FALLS, MASS.

(Read before the Connecticut Valley Dental Association.)

Ever since the days when our forefathers cut off the "tops" of teeth, burned the "nerves" out with a red-hot iron, or rammed them back up into the roots with a pine stick and inserted crowns on wooden pivots, to the present time there have been various and varied efforts to supply the loss of natural crowns with artificial substitutes, and the "end is not yet," judging from the achievements of this progressive age.

As I am assigned the task of making some "Remarks upon Bonwill's Method of Restoring Crowns upon Roots" I suppose I may choose any one of his methods, and that will be the one published in the *Dental Cosmos*, August, 1880. The first and a very important thing to do in this particular method is to get a patient who has a root needing a crown, wants it supplied, and is willing to pay for it. I shall differ with Dr. Bonwill in some particulars. Every operator who has a mind of his own and thinks for himself will not be very likely to imitate another in every particular. Each should seek to accomplish the best results in the way he can best do it.

After having settled the preliminaries, my manner of procedure is to perfectly cleanse the pulp canal and fill it from the apex down about half the length with cement or Hill's Stopping; when ready to adjust the crown, put on the rubber dam (except in some cases where the root has decayed away too far up;) remove the natural crowns with engine burs so far up that when the gum is allowed to resume its natural position it will cover the joint between the root and crown; grind the porcelain crown to conform to the root, and a little shorter than will appear when done, to allow for the amaglam between it and the root; enlarge the pulp canal sufficiently to allow for filling around the bar or pin (cutting grooves around for retainers;) take a round platinum wire as large as admissable for the hole through the crown to be used, hammer it into a square bar, make a little head on the end, barb the cornees, by trial ascertain the length required, cut off accordingly and prepare the other end the same as the one already described if not for a molar or bicuspid, but if for a cuspid or incisor flatten the end a little and barb the corners; then put bar and crown in place to see that all has been properly done; hold the bar in place in the root and fill around with amalgam as hard as can well be perfectly impacted; place around the bar and cover the base of the root with amalgam sufficiently soft to be compact with the crown; put on the crown, and with pressure and a mallet force it up into place (taking care that there is enough and not too much amalgam;) remove all surplus mercury and amalgam that may be seen; hold the crown firmly in place and fill the opening around the end of the bar with amalgam as hard as can be well worked.

When all is completed hold the crown firmly in place while pulling off the dam, and be sure the crown is kept perfectly in place till the amalgam is hard enough to retain its position. After a few days or hours the amalgam that is exposed, or that comes in contact with the gum, may be smoothly finished.

I have not seen, nor can I conceive of a case where any other course is justifiable than to fill the canal to the apex. A tap-hole is not only useless but likely to be of positive injury. The removal of the pulp and health of the periosteum should be attended to previous to the time of putting on the crown. The dam is useful, not only in keeping the operation dry but in protecting the gum and holding it away from the root. With a ligature around the root below the rubber the gum may be pressed and held up out of the way by a firm and steady pull. Where the crown is broken all off, the rubber may be adjusted by use of a clamp. Care is required in burring off the end of the root, in revolving the bur from, instead of toward the periphery. A fissure bur works very nicely for this purpose. It is better that the amalgam cover the entire end of the root rather than have the crown touch it at any point.

Hammering the wire into a square bar renders it a little stiffer, and it will be stronger than a three-cornered pin. A head on the end in the root makes it a little more secure than a tapering pin is. The head on the end in the bicuspid or molar crown should come down through, even with the grinding surface. The end in the cuspid or incisor should be flattened to correspond with the shape of the hole in the crown, and come down to the lower end of it. Pulling off the rubber has a tendency to smooth off the amalgam in the joint. Cut off no more crowns at once than can be replaced at the same sitting.

For cuspids with two roots a large wire flattened at one end and split as far as required to go into the roots, or two small wires may be used. For a bicuspid with one root the wire may be bent in the root a little. As the molar crowns have two holes they can be set very firmly.—*Dent. Miscellany.*

